# A Practical Classification System for Acute Cervical Spinal Cord Injury Based on a Three-Phased Modified Delphi Process From the AOSpine Spinal Cord Injury Knowledge Forum

**DOI:** 10.1177/21925682221114800

**Published:** 2022-09-06

**Authors:** Laureen D. Hachem, Mary Zhu, Bizhan Aarabi, Benjamin Davies, Anthony DiGiorgio, Nathan Evaniew, Michael G. Fehlings, Mario Ganau, Daniel Graves, James Guest, Yoon Ha, James Harrop, Christopher Hofstetter, Paul Koljonen, Shekar Kurpad, Rex Marco, Allan R Martin, Narihito Nagoshi, Aria Nouri, Vafa Rahimi-Movaghar, Ricardo Rodrigues-Pinto, Valerie ter Wengel, Lindsay Tetreault, Brian Kwon, Jefferson R. Wilson

**Affiliations:** 1 7938 University of Toronto, Toronto, ON, Canada; 2 Department of Neurosurgery, 1479 University of Maryland Medical System, Baltimore, MD, USA; 3 Division of Neurosurgery, Department of Clinical Neurosciences, 2152 University of Cambridge, Cambridge UK; 4 8785 University of California San Francisco, San Francisco, CA, USA; 5 Department of Surgery, 2129 University of Calgary, Calgary, AB, USA; 6 7989 University Health Network, Toronto, ON, Canada; 7 Department of Neurosurgery, 6397 Oxford University Hospitals NHS Foundation Trust, Oxford, UK; 8 6559 Thomas Jefferson University, Philadelphia, NJ, USA; 9 Department of Neurosurgery, 12235 University of Miami Miller School of Medicine, Miami, FL, USA; 10 26721 Yonsei University, Seodaemun-gu, Korea; 11 Department of Neurological Surgery, 6559 Thomas Jefferson University, Philadelphia, PA, USA; 12 7284 University of Washington, Seattle, WA, USA; 13 Department of Orthopaedics and Traumatology, 25809 University of Hong Kong, Hong Kong; 14 Department of Neurosurgery, 5506 Medical College of Wisconsin, Milwaukee, WI, USA; 15 570987 Houston Methodist Orthopedics & Sports Medicine Texas Medical Center, Houston, TX, USA; 16 Department of Neurological Surgery, 8789 University of California Davis, Sacramento, CA, USA; 17 Department of Orthopaedics, 38084 Keio University School of Medicine Graduate School of Medicine, Tokyo, Japan; 18 Department of Neurosurgery, 27230 Hopitaux Universitaires de Geneve, Genève, Switzerland; 19 48439 Tehran University of Medical Sciences, Tehran, Iran; 20 Department of Orthopaedics, 112085 Centro Hospitalar Universitario do Porto EPE, Porto, Portugal; 21 Department of Neurosurgery, 1209 Amsterdam UMC VUMC Site, Amsterdam, Netherlands; 22 12297 New York University Medical Center, New York, NY, USA; 23 Department of Orthopaedics, 8166 The University of British Columbia, Vancouver, BC, USA; 24 Department of Surgery, 10071 St Michael's Hospital, Toronto, ON, USA

**Keywords:** spinal cord injury, timing of surgery, classification system, central cord syndrome

## Abstract

**Study Design:**

A modified Delphi study.

**Objective:**

To assess current practice patterns in the management of cervical spinal cord injury (SCI) and develop a simplified, practical classification system which offers ease of use in the acute setting, incorporates modern diagnostic tools and provides utility in determining treatment strategies for cervical SCI.

**Methods:**

A three-phase modified Delphi procedure was performed between April 2020 and December 2021. During the first phase, members of the AOSpine SCI Knowledge forum proposed variables of importance for classifying and treating cervical SCI. The second phase involved an international survey of spine surgeons gauging practices surrounding the role and timing of surgery for cervical SCI and opinions regarding factors which most influence these practices. For the third phase, information obtained from phases 1 and 2 were used to draft a new classification system.

**Results:**

396 surgeons responded to the survey. Neurological status, spinal stability and cord compression were the most important variables influencing decisions surrounding the role and timing of surgery. The majority (>50%) of respondents preferred to perform surgery within 24 hours post-SCI in clinical scenarios in which there was instability, severe cord compression or severe neurology. Situations in which <50% of respondents were inclined to operate early included: SCI with mild neurological impairments, with cord compression but without instability (with or without medical comorbidities), and SCI without cord compression or instability.

**Conclusions:**

Spinal stability, cord compression and neurological status are the most important variables influencing surgeons’ practices surrounding the surgical management of cervical SCI. Based on these results, a simplified classification system for acute cervical SCI has been proposed.

## Introduction

Spinal cord injury (SCI) is a major health priority, with substantial personal and socioeconomic losses incurred by patients and caregivers.^1-3^ With the aging population, an increasing proportion of cervical injuries are now seen in elderly patients who sustain low impact falls resulting in mild or incomplete SCI.^4-6^ Compared to complete SCI, incomplete cervical injuries are more varied in their presentation in regards to associated fractures, pattern of deficits, and degree of cord compression, thus leading to a diverse range of patient phenotypes and treatment approaches.^7^ Given the heterogeneous nature of these injuries, combined with their projected increased incidence in the coming years, a clinically relevant classification scheme for cervical SCI is necessary in order to characterize disease heterogeneity and inform treatment decision making.

Classification of cervical SCI has traditionally relied on either a syndrome-based or severity-based approach. In traditional syndrome-based classification, physical examination findings have served as the main diagnostic criteria – the quintessential pattern of incomplete cervical SCI being central cord syndrome.^8^ However, it has become increasingly evident that there is significant heterogeneity among patients within a single SCI “syndrome,”^9^ thus complicating accurate assessment of natural history and treatment-associated benefits. Moreover, this framework lacks important structural measures of injury characteristics - such as fracture pattern or instability - that are essential to guide treatment decision-making in the acute care setting. In addition to syndrome-based approaches, the International Standards for Neurological Classification of SCI (ISNCSCI) have become an important tool in classifying injury severity and neurological status after SCI. However, limitations arise in using the ISNCSCI for classification, particularly in the acute setting when obtaining an exact motor or sensory score can be challenging in the face of concomitant head injury, sedation or distracting injuries. In addition, similar to syndrome-based classification, ISNCSCI does not incorporate any structural information about the integrity of the spinal column around the cord (e.g. the bones, ligaments, and disc structures).

Given the limitations of existing classification systems, development of a unifying framework to objectively classify cervical SCI, in a practical fashion, using clinically relevant parameters is necessary in order to better stratify patients into phenotypes with prognostic and therapeutic relevance.^10^ An ideal classification system must be objective, offer ease of use in the acute setting, incorporate modern diagnostic tools and provide utility in determining treatment strategies. To date, a unifying classification system that meets these priorities has yet to be established. Through a combination of expert panel discussion and an international survey of spinal surgeons, we report on the development of a new, objective, and practical classification system for cervical SCI to help facilitate communication and guide surgical decision making in the acute hospital period.

## Methods

Development of a novel classification framework for cervical SCI involved a three-phase modified Delphi method,^11,12^ which took place between April 2020 and December 2021. The modified Delphi approach allows for iterative discussions among experts in order to reach a consensus when clear supporting evidence in the field is lacking. This technique has been shown to be effective in developing clinical pathway algorithms and frameworks, and was thus well suited for the current study.^12,13^

### Three-Phase Modified Delphi Process

#### *Phase 1:* Expert Discussions on Cervical Spinal Cord Injury Classification and Management

An expert panel was established consisting of members of the AOSpine Spinal Cord Injury Knowledge Forum. This panel is comprised of 28 spinal surgeons from 10 countries, each with expertise in the diagnosis, classification and management of cervical SCI. Open ended discussions were conducted in virtual format among panel members in order to reach a consensus on a set of critical clinical variables, injury factors and imaging findings that are important for classifying cervical SCI, particularly from the perspective of deciding on the need for, and urgency of, surgical treatment for cervical SCI.

#### *Phase 2:* Assessment of Surgeon Perceptions and Practice Patterns in the Management and Classification of Cervical Spinal Cord Injury

Using the parameters of importance identified in phase 1, the expert panel developed and distributed a 22-question survey to international spine surgeons in order to assess global perceptions on the importance of these clinical variables, opinions on current cervical SCI classification systems and practice patterns in regard to cervical SCI management. The design of the survey was guided by historical surveys in the field,^14,15^ and consensus was met for the inclusion of all questions in the survey.

The first section of the survey consisted of 8 questions to obtain basic information on respondents’ demographic, practice setting and clinical experience. The second section consisted of 3 questions pertaining to the importance of specific patient, injury and imaging factors (selected by the expert panel in Phase 1) in deciding on the role and timing of surgery for cervical SCI. The third section was comprised of 8 patient scenarios with history, physical examination details and representative imaging. Respondents were asked to make a decision regarding operative or non-operative management and, if operative, the timeframe in which they would operate. These cases were developed by the expert committee to be reflective of common phenotypes of cervical SCI seen in clinical practice. Respondents were also asked 1 question pertaining to barriers they encounter when trying to expedite surgery for traumatic cervical SCI patients. The fourth section of the survey consisted of 2 questions related to respondents’ views on current classification systems of cervical SCI.

AOSpine International members (*n* = 6500) were invited by email to participate in the online survey, which was available electronically on REDCap for 30 days, with 2 reminder emails distributed. The AOSpine International membership includes a large number of spine surgeons from different countries and practice settings and is thus representative of the global spine surgery community. All surveys were completed anonymously. Descriptive statistics were used to summarize survey results.

#### Phase 3: Development of a New Classification System for Cervical Spinal Cord Injury

Phase 3 consisted of discussion among expert panel members regarding the international survey results. Panel members utilized results from the survey along with clinical expertise in order to develop a draft of a new classification system for cervical SCI. Further modifications and revisions of this classification system were done through subsequent feedback and discussions among expert panel members.

## Results

### Phase 1

Open ended discussions were conducted among experts of the panel to collate a list of the most important clinical and imaging findings that acute care clinicians use to decide on the need for, and urgency of, surgical treatment for cervical SCI. After several rounds of discussion, the following parameters were identified: presence or absence of mechanical instability, severity of neurological impairment, presence or absence of spinal cord compression, comorbidities, presence or absence of “central cord syndrome” and age.

### Phase 2: Results of International Survey

#### Demographics of Survey Respondents

**Figure 1.**
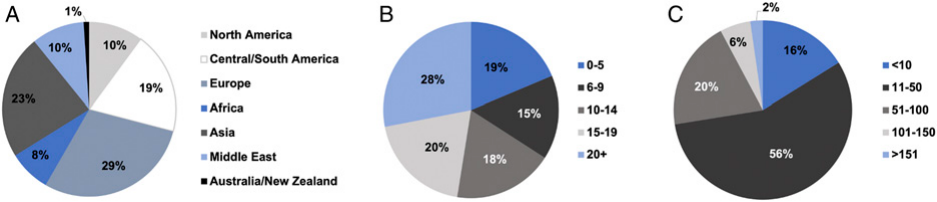
Demographics of survey respondents. (A) Respondent regional demographics, **(B)** practice experience in years, and **(C)** number of cervical trauma cases managed per year (n = 396 respondents).

A total of 396 responses were obtained, with participant demographics summarized in Figure 1A. Among respondents, 65.7% were orthopedic surgeons while 34.3% were neurosurgeons and a total of 67.2% had spine fellowship training. Sixty percent worked in a university hospital, 20.7% in a community hospital and 19.7% in private practice. Among respondents, 78.8% worked in a trauma center. The survey captured a range of respondent practice experience (Figure 1B) and exposure to cervical trauma cases (Figure 1C).

#### Perceptions on Factors Influencing Treatment Decision Making for Cervical Spinal Cord Injury

**Figure 2.**
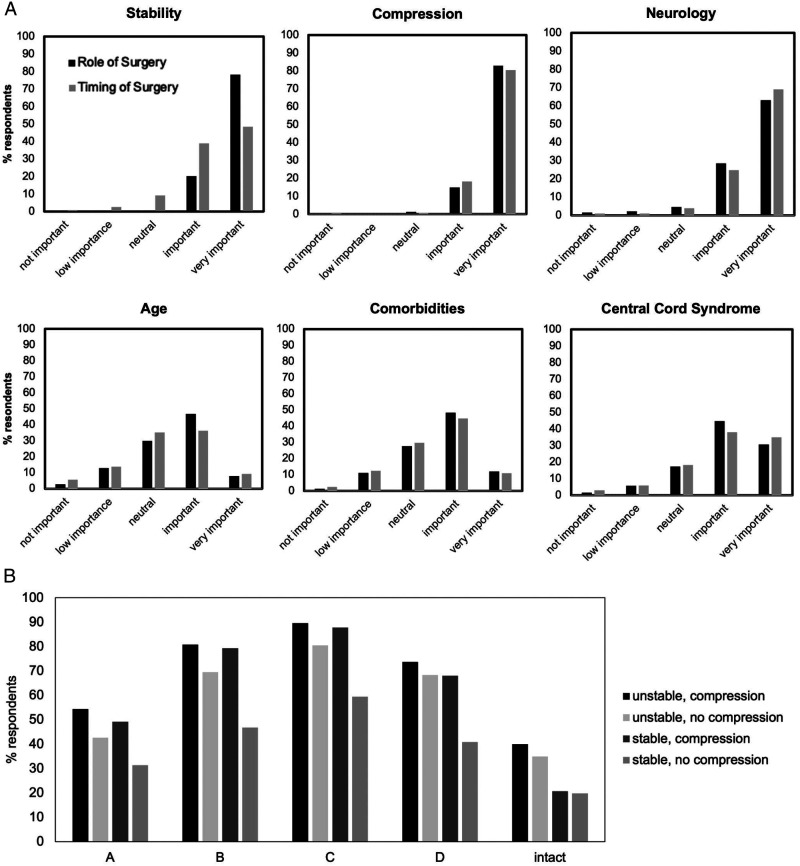
Respondent perceptions and practice patterns on surgical decision making in cervical SCI patients. (A) Respondent views on the importance of clinical parameters when deciding on the role of surgery (n = 396) and timing of surgery (n = 378) for cervical SCI. **(B)** Percentage of respondents that would perform urgent surgery (<24 hours) across various clinical contexts (n = 338 respondents).

Respondents’ perceptions on the importance of various patient and injury related factors in deciding on the role and timing of surgical intervention are shown in Figure 2A. Stability, spinal cord compression and neurological impairment were the most important factors rated by respondents when deciding on whether to operate or the timing of operation after cervical SCI. The decision to perform urgent surgery (<24 hours) across various injury patterns was strongly influenced by the AIS grade (Figure 2B). For unstable injuries (with or without compression) and stable injuries with compression, the majority of respondents would perform surgery within 24 hours in incomplete injuries (AIS B-D) with fewer respondents performing urgent surgery in motor/sensory complete injuries (AIS A).

**Figure 3.**
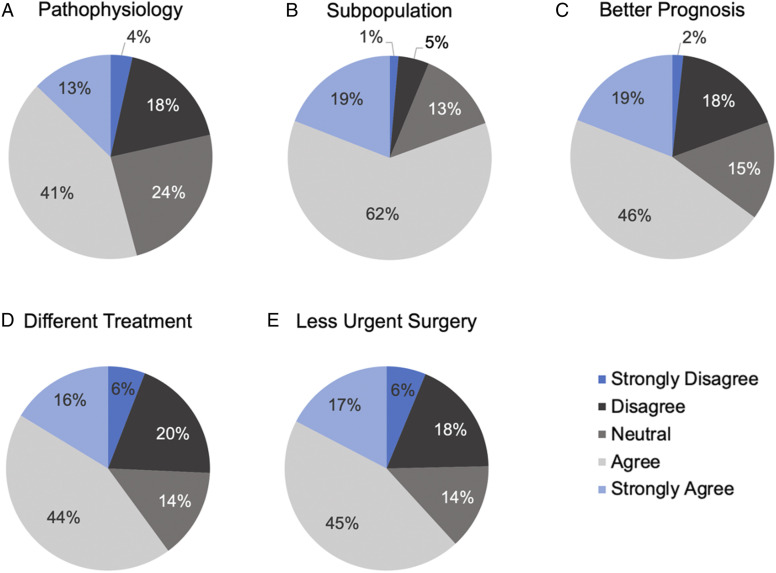
Perceptions surrounding central cord syndrome. (A, B) Respondent views on the term “central cord syndrome” in reflecting the pathophysiology of the condition and defining a subpopulation with clinical relevance. **(C-E)** Respondent views on whether the term “central cord syndrome” is associated with a better prognosis, different treatment or less urgent surgery compared to other cervical SCIs (n = 288 respondents).

Perceptions surrounding barriers to expeditious surgical intervention for traumatic cervical SCI patients were also assessed. Half (50.7%) of the respondents rated access to the operating room as a significant barrier to timely surgery, followed by availability of urgent magnetic resonance imaging/computed tomography scans (41.8%), availability of appropriate surgical personnel (38.8%), timing of paramedic transport (36.7%) and availability of perioperative intensive care unit support (27.6%). In addition, views surrounding central cord syndrome specifically were examined (Figure 3). Only 54.2% of respondents believed the term central cord syndrome accurately reflects the underlying pathophysiology of the condition and 80.6% believed it defines a subpopulation with clinical relevance. Nearly two thirds of respondents believed patients with classically defined central cord syndrome have a better prognosis than other incomplete cervical SCI patients and require less urgent surgery.

#### Practice Patterns for Cervical Spinal Cord Injury Case Scenarios

**Figure 4.**
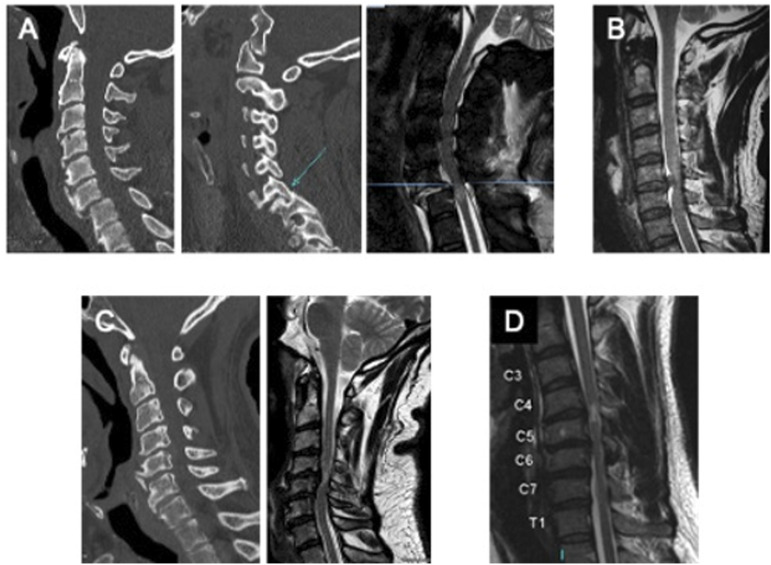
Images for case scenarios.

**Figure 5.**
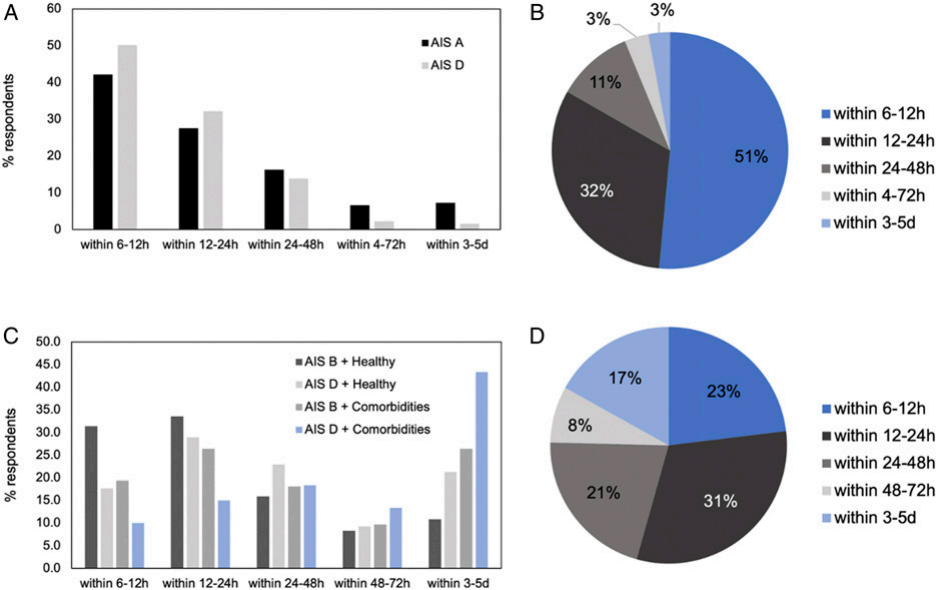
Summary of responses for the presented cases. (A) Percentage of respondents that would operate within each specified time period for scenarios 1 and 2 (n = 312 respondents). **(B)** Percentage of respondents that would operate within each specified time period in scenario 3 (n = 308 respondents). **(C)** Percentage of respondents that would operate within each specified time period for each situation in scenarios 4-7 (n = 297 respondents). **(D)** Percentage of respondents that would operate within each specified time period in scenario 8 (n = 296 respondents).

*Scenarios 1-2: A 36-year-old man involved in a diving accident sustains a C6/7 fracture dislocation. Attempts at closed reduction are unsuccessful. The associated scans are shown in* Figure 4A*.* 99.7% of respondents answered that they would operate if the patient’s neurological status was AIS grade D (severe weakness and sensory loss in both hands but otherwise preserved motor and sensory function throughout), with 82% favoring surgery within 24 hours. In the case of an AIS A patient (motor/sensory complete), 96.5% of respondents would operate, with 70% favoring surgery within 24 hours (Figure 5A).

*Scenario 3: A 27-year-old woman involved in a motor vehicle crash sustains an acute C5/6 central disc herniation. CT demonstrates no fracture. Her neurological exam is graded as an AIS C (severe motor incomplete). Her associated scan is shown in* Figure 4B*.* In this scenario nearly all respondents (99%) stated they would operate, and 83.3% would perform surgery within 24 hours, with 16.7% of patients waiting past 24 hours (Figure 5B).

*Scenarios 4-7: A 75-year-old woman with a history of cervical spondylosis sustains a hyper-extension injury following a fall down a flight of stairs. Imaging is shown in* Figure 4C*.* In the case of an AIS grade B injury (motor complete, sensory incomplete) with no medical co-morbidities, 93.6% of respondents would operate in this scenario, compared to 83.8% if the patient was AIS D (severe weakness and sensory loss in both hands but otherwise preserved motor and sensory function). The preferred time to surgery differed in these instances with more respondents favouring early surgery (<24 hours) for the more severe phenotype (65.0% for AIS B vs 46.6% for AIS D). The presence of significant medical co-morbidities reduced respondents’ preferences for the decision to operate and the time to surgery for both injury severities, however, more significantly for the milder phenotype. Specifically, for an AIS B injury, 76.7% would operate in the presence of significant medical co-morbidities and 45.8% would do so in the first 24 hours, while 26.4% would wait 3-5 days. For an AIS D injury with significant medical co-morbidities, 60.6% would operate, with only 25.0% operating within the first 2 hours, and significantly more respondents waiting 3-5d (43.3%) (Figure 5C).

*Scenario 8: A 72-year-old man has a fall from standing. His imaging demonstrates minimal cord compression but T2 signal change within the cervical cord. His neurological status is C5 AIS C (severe motor incomplete). He is otherwise healthy with no significant medical co-morbidities. His associated scan is shown in* Figure 4D*.* In this scenario, 65.9% of respondents would operate, while 34.1% would not. 54.4% would perform surgery within 24h and 16.9% would wait 3-5 days (Figure 5D).

### Phase 3: Development of a New Classification System for Cervical Spinal Cord Injury

**Figure 6.**
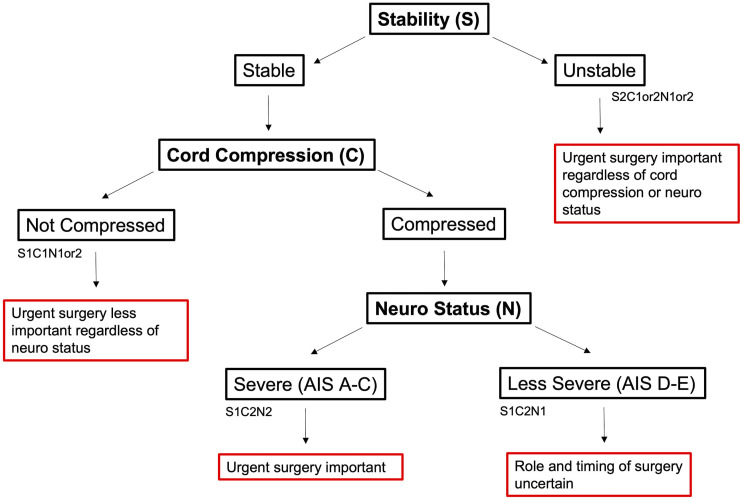
Flow diagram of proposed classification.

Results from the international survey of spine surgeons were summarized and distributed to the expert panel. From these findings, members identified and defined 3 important parameters that are critical for describing cervical injuries and are the most important factors that guide surgeon management decisions - spinal instability, cord compression, and neurological status (Table 1). On the basis of these 3 important parameters, the panel established a classification framework for cervical SCI whereby distinct phenotypes of cervical SCI with clinical relevance can be defined (Figure 6). In essence, the first factor to consider is the stability of the spinal column (S). If unstable (S2), then surgery is indicated to stabilize the spine. If stable (S1), the next factor to consider is the extent of cord compression (C). If not compressed (C1) then surgical intervention may not be required at all in the absence of instability or compression. If the cord is compressed (C2), then one considers the neurologic status next as either AIS A, B, C (severe, N1) or AIS D, E (less severe, N2). Finally, a “comorbidities modifier” was created as a summary variable accounting for patient factors that may alter surgeons’ decision making surrounding the role and timing of surgery after cervical SCI. The final classification scheme reached agreement by all panel members. The classification is described in greater detail below:

**Table 1.** Proposed Classification System of Cervical SCI.

Stability (S)	Compression (C)	Neuro status (N)	Co-morbidity modifier
Stable (1)^a^	No Compression (1)^a^	More Severe (1)	Presence of pre-existing health condition, medical frailty, medications or concomitant injuries which may modify or impact surgical treatment plan^a^
		(AIS grade A-C)	
Unstable (2)^a^	Compression (2)^a^	Less Severe (2)	
		(AIS grade D or sensory impairment only)	

^a^To be decided at the discretion of the treating surgical team.

#### Unstable

The first distinction within this classification system is between stable and unstable injuries. To allow flexibility across practices, it was decided by the expert panel that precise criteria to define stability and cord compression should be left at the discretion of the surgical team using their own standard methods for making these determinations. In patients with an unstable cervical SCI (ie S2 injuries, regardless of neural or compressive status) urgent surgery is recommended for the purposes of stabilization/re-alignment in addition to decompression depending on the presence of cord compression.^9,16^ This category encompasses all injuries in which there is concern that the structural integrity of the cervical spine has been compromised.

#### Stable, No Cord Compression

Stable SCIs can be subclassified on the basis of the presence or absence of cord compression. Stable injuries without radiographic evidence of cord compression (S1-C1-N1 or S1-C1-N2) may not require surgery at all. This subgroup may encompass injuries causing an initial impact to the cord with only transient compression.^17^ Careful work-up however should be performed in this group of patients to exclude other causes of neurological impairment (eg brachial plexus injuries or radiculopathy).

#### Stable, Cord Compression

Among patients with stable injuries and cord compression, two important subgroups may be distinguished on the basis of neurological status: patients with more severe neurological impairment (S1-C2-N2), graded AIS A-C, and patients with more mild symptoms (S1-C2-N1), graded AIS D-E.

As reflected in the survey results, patients with more severe neurologic deficits (S1-C2-N2) are considered strongly for urgent surgery with goals of decompressing the spinal cord to help facilitate improved neurological recovery.^9,16,18^ This category encompasses patients with acute traumatic disc herniations with severe impairments as well as the subset of patients who sustain a hyper-extension injury on a background of cervical spondylosis but with a more severe neurological injury. Patients with more mild neurologic impairment (S1-C2-N1) may reflect the classical “central cord injury” phenotype with mild motor/sensory deficits in the hands primarily, wherein there remains significant uncertainty surrounding the role and timing of surgical intervention.

The “comorbidity modifier” is meant as an all-encompassing term accounting for additional patient factors which may influence surgical decision making. Such factors may include pre-existing health conditions (ie cardiopulmonary disease, cancer, medical frailty), older age, medications (ie anticoagulant or antiplatelet agents) or concomitant injuries (ie traumatic brain injury, vessel injury, hemodynamic instability). Decision making surrounding the importance of such factors and the need to modify the treatment plan based on their presence is left at the discretion of the surgical team.

## Discussion

Herein we describe the development of a novel, practical classification system for cervical SCI based on a three-phase modified Delphi process incorporating a large survey gauging international surgical opinion.

Within the international survey, respondents indicated that stability of the spine, the presence of spinal cord compression and neurological status were the 3 top priorities influencing surgical decision making. It was evident that in the presence of significant spinal instability (as in scenarios 1 and 2) surgeons overwhelmingly chose to operate, most commonly within 24 hours, regardless of neurological status. However, in these scenarios, it is interesting to note that a greater proportion of surgeons wanted to proceed to surgery within 24 hours for the incomplete patient in scenario 1 (82%) as compared to the patient with complete SCI in scenario 2 (70%). In spite of several publications showing the benefits of early surgical decompression in studies including AIS grade A SCI,^9,16,19^ the current findings may demonstrate residual nihilism among spine surgeons about the prognosis of complete SCI independent of treatment offered.

A persistent area of controversy in the field of spine surgery has related to the role and timing of surgery for patients with cervical incomplete SCI with cord compression due to spondylosis but without evidence of instability, generically referred to in the past as “central cord syndrome” (Scenarios 4-7). The survey results indicate that preferences for surgical management in this context relate principally to the severity of neurological deficits seen, with surgeons more likely to operate within 24 hours in the context of an AIS grade B injury (65%) as compared to an AIS grade D injury (47%), all else being equal. In the 2010 survey by Fehlings et al, it is noteworthy that nearly an identical proportion of surgeons (roughly 50%) indicated a preference to operate within 24 hours for a similar patient scenario involving a cervical AIS grade D injury, no instability and spinal compression due to spondylosis.^14^ This indicates that although there has been an increased push towards early surgery for SCI over the last decade, including the issuing of clinical practice guidelines by AOSpine suggesting surgery within 24 hours,^9^ many surgeons remain reluctant to adopt this practice for patients with this specific injury phenotype. Although it is difficult to say with certainty, it is likely that surgeons’ reluctance in this context relates to a historically favorable view of the natural history of central cord syndrome without surgery and a paucity of modern studies specifically studying the effects of surgery in this subgroup.^20^

It is important to recognize that despite growing evidence and an increasing preference among surgeons for early surgical decompression in the setting of acute traumatic cervical SCI, a large portion of respondents identified barriers to expeditious surgical intervention which included access to operating rooms, availability of urgent imaging and surgical personnel, delays in paramedic transport and perioperative intensive care unit support. This is a practical reality that may hinder the ability to conduct surgery within a short timeframe, particularly in under-resourced settings.

In addition to stability, cord compression and neurology, other factors were important in determining the need for, and urgency of, surgery including age and comorbidities. The importance of comorbidities was highlighted in examination of surgeons’ response to patient scenarios 4-7, wherein there was less interest in proceeding with early surgery, and surgery in general, for patients with incomplete cervical SCI without instability in the setting of comorbid illness. This scenario is particularly relevant given the aforementioned shift in the epidemiology of SCI toward older patients occurring in recent years.^4-6^ Undoubtedly, surgeons will have to balance the desire to achieve neuroprotection with early surgery, with the risks of undertaking a potentially large operation in emergent fashion in frail patients with significant potential for perioperative complications. To this end, we have added a “comorbidity modifier” variable to the classification system which allows for consideration of patient or injury specific factors which may influence decision making surrounding the role and timing of surgery. As an example, in the presence of significant comorbidities or frailty, a surgeon may choose to proceed with non-operative management in the setting of a cervical SCI with a stable spine, cord compression and less severe neurological deficit (S1-C2-N1), due to concerns that the risk outweighs the potential benefit of surgical intervention.

With respect to the diagnosis of central cord syndrome, the majority of respondents still considered this to be an important diagnostic distinction with valuable prognostic and treatment related implications. However, this finding is somewhat at odds with more recent literature which provides little rationale to support continued reliance on this terminology. Specifically, there is lack of agreement surrounding what constitutes central cord syndrome; in spite of several attempts to standardize nomenclature, there remains no universally accepted definition for this diagnosis. In fact, one can identify at least 3 distinct central cord syndrome subtypes^21^ with similar neurological findings but unique injury details, including: 1) cervical incomplete injury from high impact mechanisms resulting in spinal fractures and instability; 2) cervical incomplete injury seen after a low energy mechanism trauma resulting in hyperextension injury on a background of degenerative cervical spondylosis and canal narrowing without fracture^20^, and; 3) cervical incomplete injury due to acute cervical disc herniation.^22^ While each of these situations may give rise to the prototypical central cord syndrome (disproportionate upper extremity weakness and sensory disturbance), these are obviously all very different injuries with unique treatment considerations. Apart from the lack of a standardized definition, historical concepts surrounding the underlying pathophysiology of central cord syndrome have largely been debunked, with the pattern of deficit more likely to be related to the relative preservation of extra-pyramidal motor tracts than to the theorized selective medial destruction of corticospinal tract fibers.^23-28^ Finally, from a prognostic perspective, it is noteworthy that patients with central cord syndrome have not shown to have significantly different potential for recovery as compared to cervical incomplete SCI patients without central cord syndrome.^29,30^

These points highlight that although SCI syndrome-based terminology, such as central cord syndrome, hold historical significance, it is important that more objective methods are adopted to facilitate improved communication moving forward. The proposed classification combines key objective clinical and radiological variables of vital importance necessary to describe and characterize cervical SCI and to decide on the need for, and urgency of, surgical treatment. In addition to facilitating clinical communication, it is also anticipated that the use of this classification strategy will aid in future research by allowing for clearly defined objective eligibility criteria when enrolling participants in SCI studies.

There are several limitations to this study and classification system. Namely, there was a portion of respondents who failed to respond to all survey questions. It is also acknowledged that there may be differences in opinion between surgeons specifically surrounding what constitutes a “stable” and “unstable” injury as well as what represents important spinal cord compression. While there are several systems for grading spinal stability based on fracture patterns and integrity of the posterior ligamentous complex,^31-33^ exact methods for determining stability vary between centers and surgeons due to availability of imaging tests (such as MRI) or surgeon preference. It was felt preferable by the authors to be less prescriptive in this classification allowing for some flexibility in interpretation so as not to impose definitions on surgeons with which they may or may not be comfortable. It is also important to acknowledge the possibility of conformity bias in which surgeons may provide the answer that is more socially desirable, and hence advocate more strongly for aggressive treatment (ie urgent surgery), even if their answers may not be a true reflection of their day to day clinical decision making. It will be important moving forward to complete reliability studies to more fully understand inter-rater and test-retest reliability metrics associated with this classification when applied to SCI patients.

## Conclusion

Neurological status (severe vs less severe), spinal stability and the presence of spinal cord compression appear to be the most significant variables influencing surgeons’ decision making surrounding the role and timing of surgical intervention after traumatic SCI. There is strong agreement surrounding the need for urgent surgical decompression (<24 hours) in patients with unstable injuries and spinal cord compression regardless of the severity of neurological impairments. There remains controversy surrounding the role of urgent surgical decompression in patients without spinal instability particularly in the setting of less severe neurological impairments. Based on a modified Delphi process incorporating survey findings, a simplified, practical classification system for acute cervical SCI has been proposed, which can guide management decisions.

## ORCID iDs

Laureen D. Hachem https://orcid.org/0000-0002-5886-9935

Benjamin Davies https://orcid.org/0000-0003-0591-5069

Nathan Evaniew https://orcid.org/0000-0003-1974-5224

Michael G. Fehlings https://orcid.org/0000-0002-5722-6364

Daniel Graves https://orcid.org/0000-0002-1389-5216

Paul Koljonen https://orcid.org/0000-0002-9250-653X

Rex Marco https://orcid.org/0000-0003-3393-1672

Narihito Nagoshi https://orcid.org/0000-0001-8267-5789

Aria Nouri https://orcid.org/0000-0002-4965-3059

Ricardo Rodrigues-Pinto https://orcid.org/0000-0002-6903-348X

Jefferson R. Wilson https://orcid.org/0000-0001-5965-0305
